# Optimizing Viable Leukocyte Sampling from the Female Genital Tract for Clinical Trials: An International Multi-Site Study

**DOI:** 10.1371/journal.pone.0085675

**Published:** 2014-01-15

**Authors:** Lyle R. McKinnon, Sean M. Hughes, Stephen C. De Rosa, Jeffrey A. Martinson, Jill Plants, Kirsten E. Brady, Pamela P. Gumbi, Devin J. Adams, Lucia Vojtech, Christine G. Galloway, Michael Fialkow, Gretchen Lentz, Dayong Gao, Zhiquan Shu, Billy Nyanga, Preston Izulla, Joshua Kimani, Steve Kimwaki, Alfred Bere, Zoe Moodie, Alan L. Landay, Jo-Ann S. Passmore, Rupert Kaul, Richard M. Novak, M. Juliana McElrath, Florian Hladik

**Affiliations:** 1 Department of Medicine, University of Toronto, Toronto, Canada; 2 Department of Medical Microbiology, University of Nairobi, Nairobi, Kenya; 3 Department of Obstetrics and Gynecology, University of Washington, Seattle, Washington, United States of America; 4 Department of Immunology and Microbiology, Rush University Medical Center, Chicago, Illinois, United States of America; 5 Institute of Infectious Disease and Molecular Medicine, University of Cape Town, Cape Town, South Africa; 6 Vaccine and Infectious Disease Division, Fred Hutchinson Cancer Research Center, Seattle, Washington, United States of America; 7 Department of Mechanical Engineering, University of Washington, Seattle, Washington, United States of America; 8 National Health Laboratory Services, Cape Town, South Africa; 9 Department of Medicine, University Health Network, Toronto, Canada; 10 College of Medicine, University of Illinois, Chicago, Illinois, United States of America; 11 Department of Medicine, University of Washington, Seattle, Washington, United States of America; 12 Department of Laboratory Medicine, University of Washington, Seattle, Washington, United States of America; 13 Department of Global Health, University of Washington, Seattle, Washington, United States of America; Burnet Institute, Australia

## Abstract

**Background:**

Functional analysis of mononuclear leukocytes in the female genital mucosa is essential for understanding the immunologic effects of HIV vaccines and microbicides at the site of HIV exposure. However, the best female genital tract sampling technique is unclear.

**Methods and Findings:**

We enrolled women from four sites in Africa and the US to compare three genital leukocyte sampling methods: cervicovaginal lavages (CVL), endocervical cytobrushes, and ectocervical biopsies. Absolute yields of mononuclear leukocyte subpopulations were determined by flow cytometric bead-based cell counting. Of the non-invasive sampling types, two combined sequential cytobrushes yielded significantly more viable mononuclear leukocytes than a CVL (p<0.0001). In a subsequent comparison, two cytobrushes yielded as many leukocytes (∼10,000) as one biopsy, with macrophages/monocytes being more prominent in cytobrushes and T lymphocytes in biopsies. Sample yields were consistent between sites. In a subgroup analysis, we observed significant reproducibility between replicate same-day biopsies (r = 0.89, p = 0.0123). Visible red blood cells in cytobrushes increased leukocyte yields more than three-fold (p = 0.0078), but did not change their subpopulation profile, indicating that these leukocytes were still largely derived from the mucosa and not peripheral blood. We also confirmed that many CD4^+^ T cells in the female genital tract express the α4β7 integrin, an HIV envelope-binding mucosal homing receptor.

**Conclusions:**

CVL sampling recovered the lowest number of viable mononuclear leukocytes. Two cervical cytobrushes yielded comparable total numbers of viable leukocytes to one biopsy, but cytobrushes and biopsies were biased toward macrophages and T lymphocytes, respectively. Our study also established the feasibility of obtaining consistent flow cytometric analyses of isolated genital cells from four study sites in the US and Africa. These data represent an important step towards implementing mucosal cell sampling in international clinical trials of HIV prevention.

## Introduction

Most HIV transmission occurs across a mucosal surface, especially across the mucosa of the female genital tract (FGT) [1]. In order for vaccines to prevent infection, local immunity in the cervical and vaginal mucosa is likely to be necessary. Several large HIV vaccine trials carried out in the recent past indicated that anti-HIV immune responses measured in peripheral blood may not be good surrogates of the protective efficacy elicited at mucosal sites. The HIV vaccines tested in the Step, Phambili and HVTN 505 trials failed despite stimulating strong cellular anti-HIV immune responses in peripheral blood [2]–[4], while the vaccine in the RV144 trial was marginally protective despite eliciting much weaker peripheral blood responses [5], [6]. These discrepant clinical outcomes could likely be explained by the nature of the immune responses at mucosal sites. However, mucosal sampling to assess cellular responses was not performed in these trials, mostly due to logistical challenges and a lack of knowledge about ideal sample types and processing procedures.

In addition to its importance for vaccine studies, mucosal sampling is highly relevant in microbicide trials to understand how microbicides affect the mucosa [7]–[9] and to perform pharmacodynamic studies, such as determining *ex vivo* HIV infectivity of tissue from trial participants as a surrogate of product efficacy [10], [11]. Furthermore, mucosal sampling is integral to studies of the basic immunobiology of the FGT and of other sexually transmitted infections. Diverse methods exist for sampling cells from the FGT, including most prominently cervicovaginal lavage (CVL), endocervical cytobrushes and ectocervical biopsies. The relative cellular yield from these procedures is unclear, as are any differences in the leukocyte subpopulations obtained from each procedure. Here we address the question of optimal mucosal sample type in an international, multisite collaboration. We find that CVL is unsuitable for cellular analysis, while two sequential cervical cytobrushes give a similar number of leukocytes (about 10,000 cells) to one biopsy, though the subpopulation profiles of the isolated cells differ. Our results provide guidance for mucosal cell sampling, processing, and flow cytometric analyses in HIV prevention trials.

## Methods

### Participant characteristics and study sites

Four research sites participated in the study: Chicago, USA; Nairobi, Kenya; Cape Town, South Africa; and Seattle, USA. The Institutional Review Boards at each site approved the study (University of Illinois at Chicago, Kenyatta National Hospital, University of Cape Town, University of Washington). All main study participants gave informed written consent prior to enrollment. Women between ages 18 and 55 were eligible for the study if they were HIV uninfected, tested negative for gonorrhea, chlamydia, and trichomonas at the sampling visit, and reported at least six normal menstrual cycles within the past year (except in South Africa where a number of women using the hormone contraceptive depot medroxyprogesterone acetate [DMPA] were amenorrheic). At the time of each study visit, blood was obtained for HIV testing by ELISA, first-catch urine for gonorrhea and chlamydia testing by nucleic acid amplification, and vaginal swabs from the posterior fornix for Trichomonas testing by Inpouch™ culture (BioMed Diagnostics, White City, OR, USA) and for Gram staining to diagnose bacterial vaginosis (BV) by Nugent scoring. Gram stains with a Nugent score ≥7 or a Nugent score of 4–6 and the presence of clue cells were classified as signifying BV. BV was not an exclusion criterion. Hormonal contraception use differed between participants and sites, as described in the Results.

At the Seattle site, discarded vaginal tissues from vaginal repair surgeries were used for optimization of the biopsy processing method and development of the α4β7 staining protocol. These tissues were obtained anonymously under a waiver of consent approved by the IRBs of the University of Washington and the Fred Hutchinson Cancer Research Center.

### Sample collection and transport

Samples were collected between 16–24 days from start of the previous menstrual cycle (first day of menstruation), which is considered to be a window of vulnerability for HIV infection [12], [13]. All samples were transported to the laboratories on wet ice and processed within four hours of collection.

In Part 1 of the study, we compared the immune cells obtained by endocervical cytobrush and CVL at three study sites, Chicago, Nairobi and Seattle. Different sites for this part of the study used different cytobrush samplers according to their standard collection method. Chicago used Cytobrush Plus GT cell collectors (CooperSurgical, Trumbull, CT, USA), Nairobi used Bio Nuclear Diagnostics CYT-003A Endocervical cytobrushes, and Seattle used Cytobrush Plus cell collectors (CooperSurgical). Samples were obtained in the following order: diagnostic samples for STI testing, CVL, and cytobrush. CVL was obtained by placing 10 mL of phosphate-buffered saline on the cervix and collecting the fluid from the posterior fornix. Collected fluid was placed in a 15 mL conical tube. For the cytobrush samples, mucus was wiped from the cervix, the cytobrush was gently inserted into the cervical os, rotated 360°, removed, and placed into 5 mL RPMI-1640. A second cytobrush immediately followed. Any visible blood contamination was noted.

In Part 2 of the study, we compared endocervical cytobrush and ectocervical biopsy sampling at three study sites, Chicago, Nairobi and Seattle. In this part of the study, all sites used Digene Cervical Sampler cytobrushes (Qiagen, Valencia, CA, USA). Sampling for Part 2 occurred in the following order: diagnostic samples for STI testing, cytobrush, and biopsy. Cytobrushes were collected as above. One biopsy was taken from the upper left quadrant of the ectocervix using a Baby Tischler Biopsy Forceps (Wallach Surgical, Trumbull, CT, USA) with a 4.2×2.3 mm bite size. A second biopsy was taken from the upper right quadrant in a subset of participants from Nairobi. The biopsy was placed into 5 mL RPMI-1640. No local injection anesthetic was used before biopsy collection.

Endocervical cytobrushes were also obtained in Cape Town from women with or without DMPA contraception, using the Digene Cervical Sampler cytobrushes. Cape Town did not perform CVL or biopsy sampling.

### Cervicovaginal lavage (CVL) processing

Samples were centrifuged and the pellet transferred into tubes for flow cytometric staining. The full CVL processing protocol is included in the File S1.

### Endocervical cytobrush processing

The two cytobrushes were processed sequentially and the extracted cells were combined for staining and flow cytometric analysis. Cytobrushes were inserted into 25 mL serological pipettes containing 20 mL room temperature phosphate buffered saline (PBS). The brush was moved in and out of the tip of the pipette while the PBS was gradually expelled, extracting the cells from the cytobrush and washing them through a 100 µm cell strainer into a 50 mL conical tube (BD, Franklin Lakes, NJ, USA). This was repeated with another 10 mL PBS and then the cytobrushes were scraped on the edge of the cell strainer and discarded. The 5 mL RPMI-1640 in the transport tube was passed through the strainer and the tube washed with an additional 15 mL PBS. If the strainer became clogged with mucus, the mucus was pipetted up and down in the serological pipette and broken up. After straining, the 50 mL cell suspension was pipetted up and down several times to break up any remaining mucus. The full cytobrush processing protocol is included in File S2.

### Leukocyte isolation from ectocervical biopsies

To determine the optimal method of leukocyte isolation from biopsies, three methods were tested on human vaginal tissue: collagenase digestion, emigration, and enzyme cocktail digestion. Emigration and collagenase digestion were based on prior experience in the Seattle laboratory [14], [15]. The enzyme cocktail digestion was based on a protocol used in the laboratory of Dr. Craig Hendrix in Baltimore [16]. The methods were tested in parallel, with three replicates each time, on tissue from two separate donors. The cells were stained with the panel described below and cell yield and distributions were compared.

### Leukocyte isolation by collagenase digestion

R15 and collagenase digestion media were prepared fresh before each biopsy digestion. R15 consisted of RPMI-1640, penicillin-streptomycin and L-glutamine (Gibco), supplemented with 15% heat inactivated fetal bovine serum (Gemini Bio-Products, West Sacramento, CA, USA). Collagenase digestion media consisted of collagenase type II (Sigma-Aldrich C6885, St. Louis, MO, USA) dissolved at 1 mg/mL (693 collagen units or 2.8 FALGPA units per mL) in a 1∶1 mixture of PBS and R15, based on a titration in which we determined 700 collagen units/mL to be optimal. All sites used the same lot of collagenase (091M8608V). Collagenase digestion media was warmed for 30 minutes in a 37°C water bath before use. On receipt in the lab, biopsies were washed with 10 mL PBS and in some instances, to facilitate digestion, cut into smaller pieces with sterile razor blades. Samples were then placed into an 8 mL round-bottom tube (BD) containing 3 mL collagenase digestion media, with 1 unit/mL DNase 1 (Sigma) to prevent cell clumping. The tubes were shaken at 200 rpm in a 37°C incubator for 30 minutes. The collagenase digestion media and biopsies were then aspirated into and expelled from a 3 mL syringe through a blunt 16 gauge needle ten times and the cell suspension passed through a 70 µm cell strainer into fresh R15. The tube, syringe, and strainer were then washed with R15, which was added to the cell suspension. The tissue collected on the strainer was placed in fresh collagenase digestion media with DNase and shaken for another 30 minutes. This cycle was repeated up to four times. Any material left after the fourth digestion was discarded. During subsequent rounds of digestion, the cell suspension was centrifuged and cell pellets were resuspended in R15 and kept on ice. The full collagenase digestion protocol is included in File S3.

### Leukocyte isolation by emigration

Biopsies were washed five times with cold PBS in a 15 mL tube, placed in RPMI-1640 in a well of a six-well plate, and cut into small pieces with sterile razor blades. Cold PBS was added vigorously and then the supernatant removed carefully ten times to wash away loose epithelial cells and decrease the chance of bacterial or fungal contamination. After the tenth wash, 5 mL R10 (RPMI-1640, penicillin-streptomycin and L-glutamine (Gibco), supplemented with 10% heat inactivated fetal bovine serum) was very gently added and the biopsies cultured in a 37°C incubator with 5% CO_2_ for 48 hours. After incubation, collagenase D (Applied Science, Indianapolis, IN, USA) was added drop-wise to the wells for a final concentration of 2 mg/mL and incubated for 10 minutes at 37°C. The supernatant containing the cells was then carefully removed without disturbing the tissue. The biopsies were washed very gently with media and the media combined with the cell suspension.

### Leukocyte isolation by enzyme cocktail

Biopsies were digested as in the collagenase protocol, with the following modifications. The same collagenase was used at the same concentration, with the addition of Elastase at 0.07 U/mL (Worthington Biochemicals Lakewood, NJ, USA), Hyaluronidase at 0.4 U/mL (Worthington), and DNase I 0.083 U/mL (New England Biolabs, Ipswich, MA, USA). The enzymes were dissolved in R7.5 (RPMI-1640, penicillin-streptomycin and L-glutamine (Gibco), supplemented with 7.5% heat inactivated fetal bovine serum) rather than a mixture of R15 and PBS. Biopsies were vortexed in digestion media for 1 min and then shaken for 30 minutes. After shaking, the cells were passed through a strainer and the tissue pieces broken up with a 200 µL pipet tip. Biopsies were then returned to the shaker in fresh digestion media. Digestions were repeated a total of up to three times.

### Staining and flow cytometry acquisition

Cells suspended in PBS were transferred to 5 mL round-bottom, snap cap tubes (BD) and stained with the LIVE/DEAD Fixable Aqua Dead Cell Stain kit (Life Technologies, Grand Island, NY, USA) in PBS. Cells were washed with FACS wash (PBS containing 1% bovine serum albumin [Sigma]) and stained with the antibody panel for phenotyping (Table S1). Cells were washed again with FACS wash, resuspended in 250 µL 1% paraformaldehyde, and transferred to Trucount Absolute Counting Tubes (BD). The staining tubes were washed with an additional 250 µL 1% paraformaldehyde, which was then added to the Trucount tubes. In Seattle and Nairobi, cells were stained in v-bottom, 96-well plates for Part 1; all sites used tubes in Part 2.

In Part 2, after the viability stain, cells were stained with Act-1 [17], washed and stained with biotinylated goat anti-mouse antibody (Beckman Coulter, Brea, CA, USA), washed twice and stained with the phenotyping panel plus streptavidin PE-Cy5 (BD). Antibody panel optimization and titrations were performed in peripheral blood mononuclear cells, followed by confirmation using cells isolated from cytobrushes. The Act1 reagents were titrated using digested vaginal tissue obtained from vaginal repair surgeries.

Compensation controls were prepared simultaneously with sample processing, using CompBead Plus (BD) for antibodies and ArC Amine Reactive Compensation Beads (Life Technologies) for the viability stain. Samples were acquired on LSRII flow cytometers (BD), equipped with 405 nm, 488 nm, and 635 nm lasers at all sites, with the addition of a 535 nm laser in Seattle. Forward and side scatter voltages were normalized using Trucount beads and fluorescence parameter PMTs were normalized by use of Rainbow Calibration Particles, Peak 7 (Spherotech, Lake Forest, IL, USA).

### Data analysis

Standardization of procedures and assays enabled data pooling and comparison between laboratories at multiple sites. FACS data files were transferred to the Seattle site for centralized analysis, which was done by co-author SMH using FlowJo 9.6 for Mac (Tree Star, Ashland, OR, USA), Excel 2010 for Windows (Microsoft, Redmond, WA, USA), and Prism 5.01 for Windows (GraphPad Software, La Jolla, CA, USA). The gating scheme is shown in Figure S1. Absolute cell numbers were determined by measuring the number of Trucount beads acquired and dividing this number by the known total number of beads in the tube, thus determining the fraction of the sample acquired. From there, the number of cells acquired was divided by the fraction of the sample acquired to calculate the total number of cells present in the sample.

The α4β7 analysis only included samples where at least 50 cells of a given subset were recovered. The positive staining gate was set by using the CD45^neg^ cell population, which we had determined in preliminary experiments to match FMO controls, as an internal negative control. The general cut-off for α4β7 positivity was set at the threshold of 99.5% of CD45**^neg^** cells staining α4β7 negative. Due to non-specific binding of the streptavidin PE-Cy5 reagent to macrophages, no information about α4β7 expression on these cells could be determined.

### Statistical analyses

*Sample sizes were calculated to detect differences between the three sample types in the primary outcome measurement of total mononuclear leukocyte yields within each study site.* Wilcoxon signed rank tests were used to compare two different sample types from the same participants (Parts 1 and 2) and to compare the same sample types from participants collected at different time points. Inter-site variations of cell numbers and frequencies were tested using unpaired Kruskal Wallis ANOVA (with Dunn's multiple comparison adjustment). Mann Whitney tests were used to compare the effect of visible red blood cells, and of DMPA use, on cell yields. All tests were two-tailed at α = 0.05. Pearson correlations were used to analyze the linear association between cytobrush sampling at two different visits and between duplicate biopsies.

## Results

### Participant characteristics

This study was conducted in two parts and enrolled women from four study sites. The participants were HIV-uninfected healthy women between the ages of 18 and 37. In Part 1 of the study (comparison of CVL and cervical cytobrush), we enrolled 16 women each from Chicago (median age 25, IQR 24–29), Nairobi (31, 27–34) and Seattle (32, 27–37) for CVL and endocervical cytobrush sampling. In Part 2 (comparison of cervical cytobrush and cervical biopsy), we enrolled 17 women from Chicago (27, 23–30) and Nairobi (25, 23–27), and 20 from Seattle (30, 27–36), for cytobrush and cervical biopsy sampling. In addition, we enrolled 25 women from Cape Town for cytobrush sampling only (median age 31, IQR 26–33). Five additional women tested positive for an STI in the screening phase and were therefore not enrolled. One additional Seattle participant was excluded because the biopsy was not kept on ice and one additional Chicago participant was excluded for amenorrhea.

### Part 1: Comparison of endocervical cytobrushes and cervicovaginal lavages

**Figure 1:**
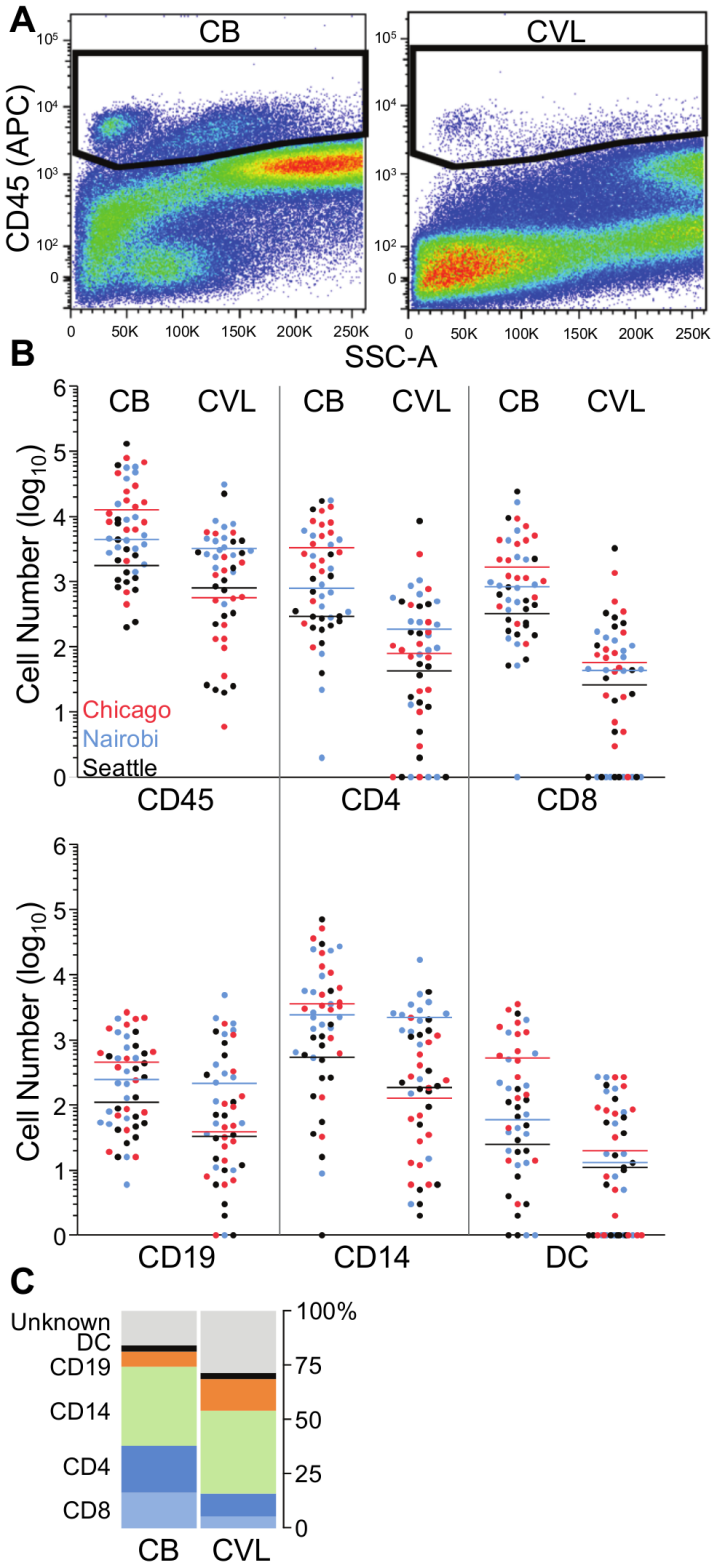
Comparison of immune cell yield and distribution in endocervical cytobrush (CB) and cervicovaginal lavage (CVL) samples. (**A**) Representative gates for identification of CD45^+^ leukocytes from cytobrush and CVL samples. (**B**) Numbers of CD45^+^ leukocytes, CD4^+^ T cells, CD8^+^ T cells, CD19^+^ B cells, CD14^+^ macrophages, and CD19^neg^/HLA-DQ^+^ DC enumerated from cytobrush and CVL samples. Cell numbers were log_10_ transformed, and plotted per site (Chicago, red dots; Nairobi, blue dots; Seattle, black dots). Horizontal bars indicate median values for each site. (**C**) Percentage contribution of each cell subset, as well as of cells that do not fit one of the described populations (“unknown”), to the total CD45^+^ population from cytobrush and CVL samples. Data are averaged across sites. P values are listed in the text, and median and IQR in Table 1.

In Part 1, carried out in Chicago, Nairobi and Seattle, we compared the yield and composition of leukocytes obtained from two sequential endocervical cytobrushes to one 10 mL CVL from each participant. Subpopulations of CD45^+^ leukocytes were phenotyped using a pre-defined gating strategy (Figure S1), and the absolute number of viable cells per sample was calculated using the fraction of absolute counting beads acquired. Cytobrush sampling yielded more viable leukocytes than CVL (Figures 1A and B). The median number of viable CD45^+^ leukocytes from two sequential cytobrushes was 5,810 (IQR 2,075–17,656) across all sites, while CVL samples yielded a median of 1,667 CD45^+^ cells (IQR 321–4,185; p<0.0001). The higher CD45^+^ cell yield from cytobrushes was observed at all sites (p<0.05 in Chicago and Nairobi; p = 0.11 in Seattle). Of note, only a minority of all cells obtained by either method expressed CD45, with the percentage of CD45^+^ cells significantly higher in cytobrush versus CVL (cytobrush median 0.81%, IQR 0.27–1.7; CVL median 0.09%, IQR 0.03–0.24; p<0.0001). The viability of CD45^+^ leukocytes was similar between the two sample methods (cytobrush median 68.9%, IQR 56.3–82.9; CVL median 67.1%, IQR 36.6–93.5; p = 0.6779).

Consistent with larger overall CD45^+^ populations, cytobrushes yielded significantly more CD4^+^ and CD8^+^ T cells, CD14^+^ macrophages, CD19^+^ B cells, and CD19^neg^ HLA-DQ^+^ DCs than CVLs (Figure 1B and Table 1). Macrophages/monocytes were the largest subpopulation in both sample types (medians of 2,233 for cytobrush and 266 for CVL; p = 0.0001). T cells were the second largest population (medians 1,170 and 89 for CD4^+^; 815 and 44 for CD8^+^; both p<0.0001). B cells and dendritic cells (DC) were much less frequent (medians of 236 and 59 for B cells; p = 0.044; 83 and 11 for DCs; p<0.0001). Statistical significance was not always observed when the analysis was repeated on individual site level data, but the directions of the trends remained the same.

**Table 1:** Median (IQR) numbers of leukocyte subpopulations in endocervical cytobrushes (CB) and cervicovaginal lavages (CVL).

	Chicago		Nairobi		Seattle	
	CB	CVL	CB	CVL	CB	CVL
CD45^+^	12,956 (6,502–29,177)	593 (144–2,347)	4,869 (3,261–32,486)	3,235 (2,449–6,386)	1,772 (869–7,037)	801 (76–2,809)
CD4	3,350 (1,563–8,108)	82 (13–222)	798 (299–4,288)	185 (29–553)	294 (202–1,204)	44 (7–356)
CD8	1,689 (700–4,399)	55 (10–153)	840 (194–2,339)	45 (0–101)	322 (162–781)	26 (0–225)
B	453 (76–1,700)	49 (20–138)	214 (56–704)	245 (40–1370)	111 (43–531)	33 (7–248)
MΦ	3,641 (1,611–13,081)	145 (25–558)	2,434 (1,490–8,660)	2201 (401–3,333)	541 (76–1,579)	187 (17–1187)
DC	545 (72–1,550)	21 (0–94)	59 (15–429)	13 (0–143)	25 (3–107)	11 (0–50)

CD4, helper T lymphocytes; CD8, cytotoxic T lymphocytes; B, B lymphocytes; MΦ, macrophages; DC, dendritic cells.

In addition to absolute numbers, we evaluated the percentage of the CD45^+^ compartment constituted by each subpopulation (Figure 1C). T lymphocytes were relatively enriched in cytobrush compared to CVL (median 20.4 vs. 6.8% for CD4; 15.8 vs. 3.9% for CD8; p<0.0001 for both T cell subsets), whereas the reverse was seen for B cells (3.5 vs. 7.6%; p = 0.0017). No differences were observed in the percentages of macrophages or DCs between cytobrush and CVL (35.6 vs. 34.2% for macrophages; 2.1 vs. 2.0% for DCs; p = 0.8335 and p = 0.7779, respectively). Cytobrushes contained a substantially lower fraction of CD45^+^ cells than CVL that could not be defined as one of the five immune subpopulations (median 14.5 vs. 27.5%; p = 0.0005; Figure 1C).

In summary, two sequential cytobrushes give substantially greater numbers of all leukocyte populations tested than CVL samples. We therefore chose cytobrush sampling as the better of the two non-invasive methods to compare to biopsy sampling in Part 2 of the study.

### Part 2: Comparison of endocervical cytobrushes and ectocervical biopsies

Mucosal biopsies are traditionally believed to be the “gold standard” for obtaining the highest cell yields. However, biopsy sampling is invasive and we were therefore interested to systematically evaluate whether comparatively non-invasive cytobrush sampling yields equivalent viable cell numbers and distributions of leukocyte subpopulations.

As a preparatory step for Part 2, we first determined the optimal method for leukocyte isolation from cervical tissue. We compared three methods using equally sized biopsies taken from vaginal tissue obtained from several patients undergoing vaginal repair surgeries. Collagenase plus DNase digestion yielded substantially greater numbers of CD45^+^ leukocytes (median 119,248) than either a cocktail of four enzymes (median 25,136) or emigration (median 21,588) (p<0.05; Figure S2A). Cells obtained by emigration were proportionally enriched for CD45 expression, although overall numbers were lower, and the cells obtained by this method were almost exclusively T cells. T cells were also the dominant leukocyte population isolated by collagenase or enzyme cocktail digestion, but both enzyme digestion procedures gave substantial frequencies of CD14^+^ macrophages, few of which were seen in the emigration protocol (Figure S2B). Due to the substantially larger CD45^+^ yield from the collagenase digestion protocol, this procedure was chosen for the formal comparison with cytobrushes in Part 2. All three participating sites (Chicago, Nairobi and Seattle) used the same lot and concentration (1 mg/mL or 693 collagen units/mL) of collagenase, which we determined to be optimal for cell yield without reducing expression of surface antigens (Figures S2C and D).

**Figure 2:**
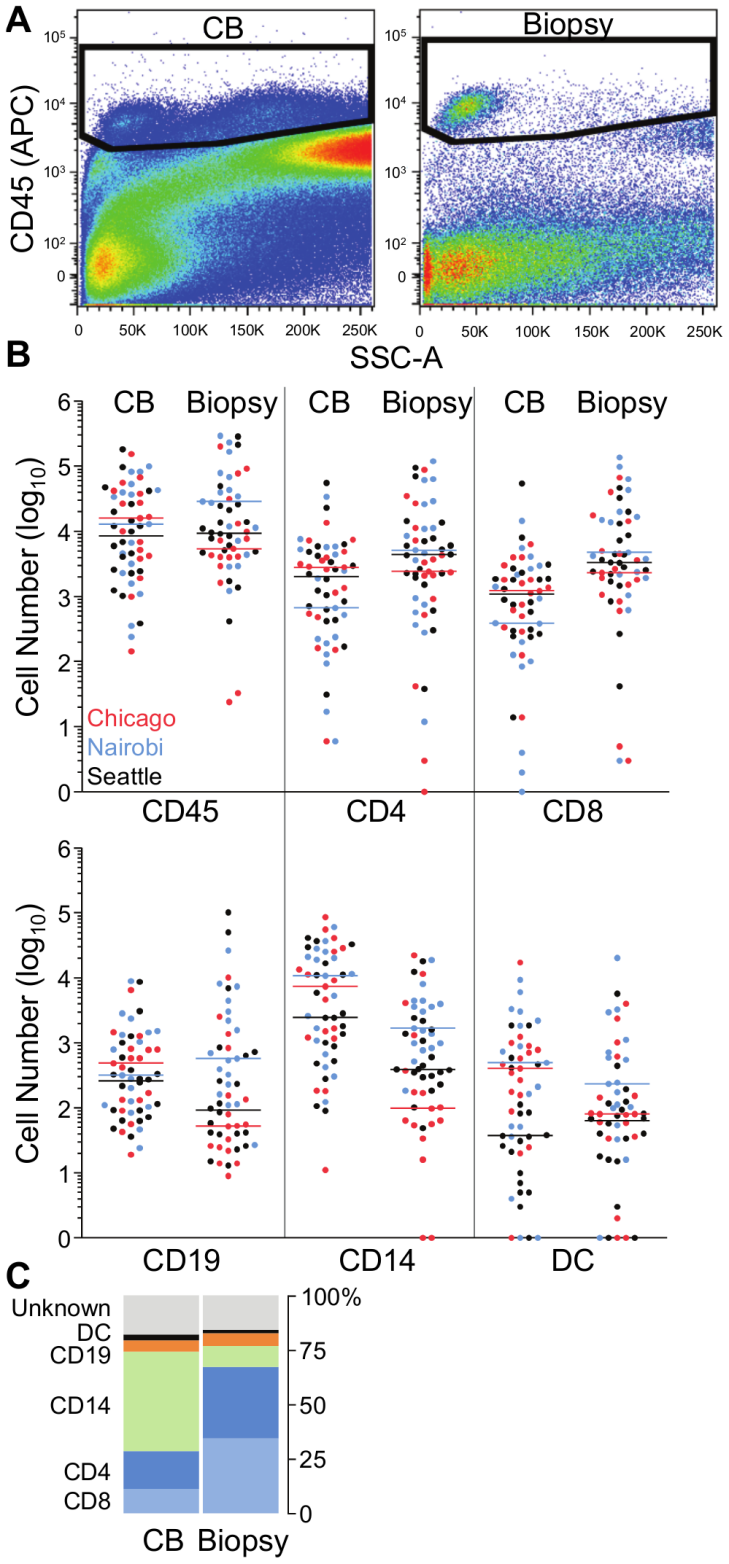
Comparison of immune cell yield and distribution in endocervical cytobrush (CB) and ectocervical biopsy samples. (**A**) Representative gates for identification of CD45^+^ leukocytes from cytobrush and biopsy samples. (**B**) Numbers of CD45^+^ leukocytes, CD4^+^ T cells, CD8^+^ T cells, CD19^+^ B cells, CD14^+^ macrophages, and CD19^neg^/HLA-DQ^+^ DC enumerated from cytobrush and biopsy samples. Cell numbers were log_10_ transformed, and plotted per site (Chicago, red dots; Nairobi, blue dots; Seattle, black dots). Horizontal bars indicate median values for each site. (**C**) Percentage contribution of each cell subset, as well as of cells that do not fit one of the described populations (“unknown”), to the total CD45^+^ population from cytobrush and biopsy samples. Data are averaged across sites. P values are listed in the text, and median and IQR in Table 2.

We then proceeded to Part 2 of our study, carried out in Chicago, Nairobi and Seattle. We found that two sequential, combined Digene cytobrushes yielded comparable numbers of CD45^+^ leukocytes as one 4.2×2.3 mm bite size biopsy (cytobrush median 11,008, IQR 2,396–36,873; biopsy median 9,717, IQR 4,092–25,535; p = 0.53) (Figures 2A and B, Table 2). Viable CD45^+^ cells made up a smaller percentage of all cells in cytobrush than in biopsies (cytobrush median 1.0%, IQR 0.43–2.6; biopsy median 4.5%, IQR 0.97–11.3; p<0.0001), indicating that fewer non-immune cells are present in biopsy-derived samples. This was consistent across the sites (all p<0.05). Leukocytes from cytobrushes had slightly lower viability than from biopsies (median 76.1%, IQR 63.0–87.1 vs. 84.4%, IQR 70.0–89.9; p = 0.0010); this was consistent in Nairobi and Seattle, but not observed in Chicago (p = 0.7764).

**Table 2:** Median (IQR) numbers of leukocyte subpopulations in endocervical cytobrushes (CB) and ectocervical biopsies.

	Chicago		Nairobi		Seattle	
	CB	Biopsy	CB	Biopsy	CB	Biopsy
CD45^+^	15,893 (4,018–39,742)	5,345 (3,467–22,357)	12,942 (1,930–48,080)	28,645 (5,754–73,043)	8,396 (2,471–24,604)	9,369 (4,935–23,644)
CD4	2,797 (515–4,407)	2,422 (721–9,287)	672 (161–5,496)	5,117 (659–27,530)	2,014 (649–4,687)	4,341 (2,006–7,623)
CD8	1,229 (418–2,404)	2,310 (949–9,608)	387 (93–2,820)	4,753 (2,050–31,614)	1,099 (300–2,204)	3,303 (1,835–8,327)
B	485 (112–801)	53 (24–492)	317 (107–1,478)	571 (196–3,738)	261 (77–543)	92 (39–701)
MΦ	7,380 (1,193–27,016)	98 (44–845)	10,814 (875–23,052)	1,683 (913–4,206)	2,445 (616–15,326)	392 (300–1,550)
DC	405 (123–842)	81 (35–386)	495 (44–2,625)	234 (77–916)	37 (8–337)	63 (17–111)

CD4, helper T lymphocytes; CD8, cytotoxic T lymphocytes; B, B lymphocytes; MΦ, macrophages; DC, dendritic cells.

Cytobrushes and biopsies differed considerably in the distributions of leukocyte subpopulations (Figure 2B and C). Cytobrushes yielded notably fewer T cells than biopsies, with lower levels of both CD4^+^ (median 2,043 vs. 3,979; p = 0.0044) and CD8^+^ T cells (median 1,207 vs. 3,472; p<0.0001). Similarly, both T cell subsets made up smaller fractions of CD45^+^ cells in cytobrushes than in biopsies (median 15.7 vs. 36.3% for CD4; 8.1 vs. 35.8% for CD8; both p<0.0001). Conversely, cytobrushes contained a higher number and proportion of CD14^+^ cells compared to biopsies (median 4,975 vs. 559; median 45.6 vs. 4.5% CD14^+^ cells; both p<0.0001). Both sample types contained small numbers of B cells and DCs, with cytobrushes having about twice the number of both CD19^+^ B cells (298 vs. 146; p = 0.9143; 3.6 vs. 1.9%; p = 0.1409) and DCs (171 vs. 82; p = 0.0835; 1.9 vs. 1.2%; p = 0.0004) when compared to biopsies. CD45^+^ leukocytes isolated from cytobrushes contained a modestly greater percentage of cells than biopsies that could not be defined as one of the five immune subpopulations (15.8 vs. 12.1%; p = 0.1409) (Figure 2C). Therefore, while no overall difference in CD45^+^ cell recovery was observed between sample types, there were significant differences in terms of composition, with cytobrushes containing relatively more macrophages and biopsies more T cells.

### Inter-site variation

**Figure 3:**
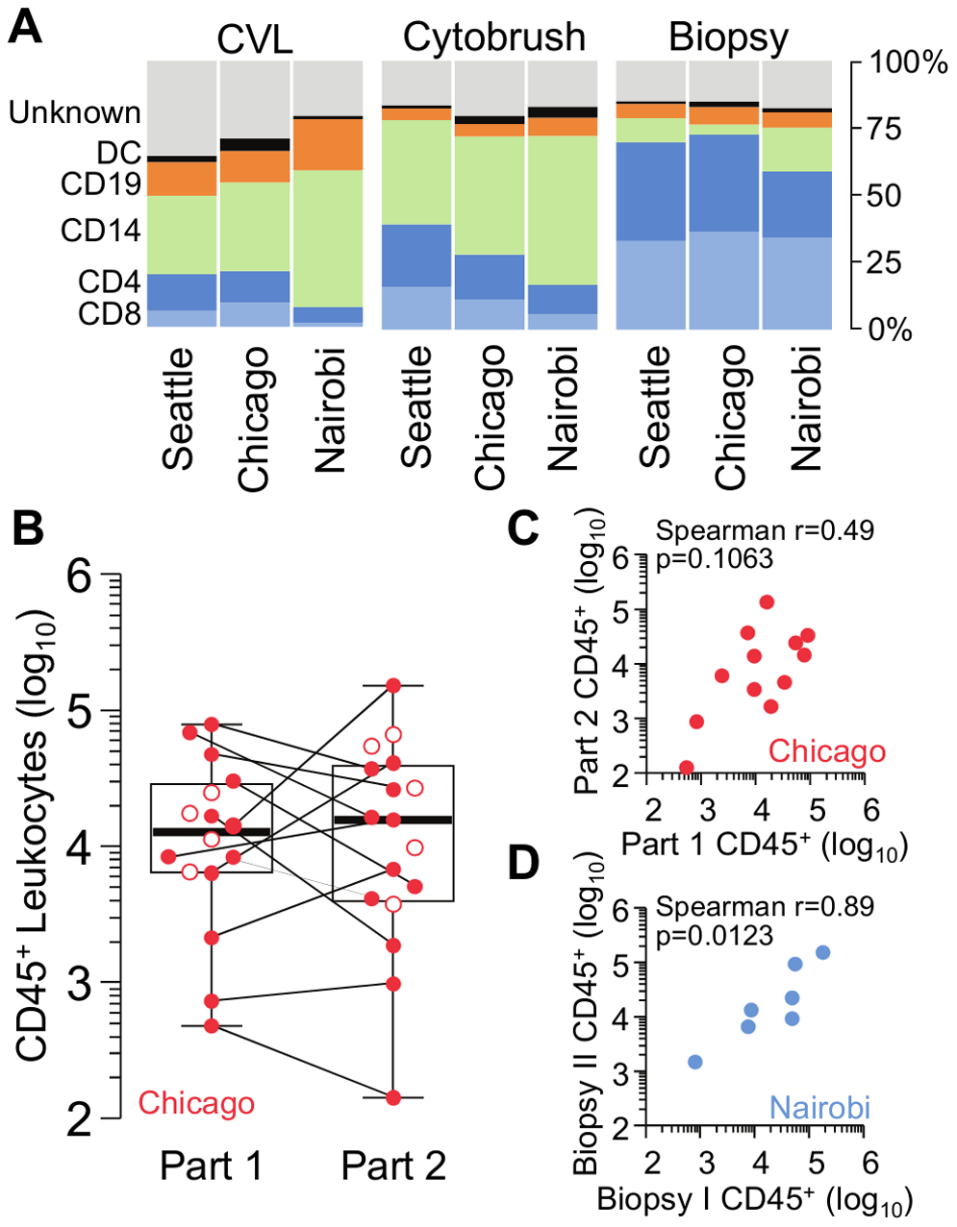
Inter-site variability and sample reproducibility from female genital tract immune cell sampling techniques. (**A**) Percentage contribution of each immune cell population to the total CD45^+^ population recovered from CVL, cytobrush, and biopsy samples collected at the Seattle, Chicago, and Nairobi study sites. (**B**) Numbers of CD45^+^ leukocytes recovered from cytobrush samples collected from all women recruited in Chicago. Filled circles and connecting lines indicate women participating in both Part 1 and Part 2 of the study (n = 12). Open circles indicate women sampled only in one part of the study. Boxes indicate median, interquartile range and total range. (**C**) Scatterplot of cell numbers from the twelve women sampled in both Part 1 (x axis) and Part 2 (y axis) of the study. (**D**) Scatterplot of cell numbers from replicate biopsies collected at the same clinic visit in seven participants at the Nairobi study site. Biopsies were taken from upper left and upper right quadrants of the ectocervix.

To evaluate inter-site variation, we compared the recovery of CD45^+^ leukocytes and each leukocyte subpopulation between the Chicago, Nairobi and Seattle study sites (Figure 3). For CVL samples, the main difference was that samples from Nairobi contained significantly more CD45^+^ cells than those from Chicago and Seattle (p<0.05 and p<0.01), which was possibly driven by increased numbers in CD14^+^ cells (both p<0.05). Chicago and Seattle CVLs also had proportionally fewer CD8^+^ T cells than Nairobi (p<0.001 and p<0.01), and Chicago had proportionally more DCs than Nairobi (p<0.05) (Figure 3A, left).

Cytobrush samples were remarkably consistent across sites, especially in Part 2, where all sites were more experienced and used the same device. In Part 2, there were no statistically significant differences in the total number of CD45^+^ cells or leukocyte subpopulations between the sites, but there were some differences in the frequencies of certain subpopulations (Figure 3A, center). The percentages of CD4^+^ and CD8^+^ T cells were higher in Seattle than in Nairobi (both p<0.01). Samples from both Nairobi and Chicago contained larger percentages of dendritic cells than those from Seattle (p<0.01 and p<0.05).

Biopsy samples were also relatively consistent across sites (Figure 3A, right). Some minor differences were noted, specifically in the number of DCs (Nairobi having more than Seattle; p<0.05), the number of B cells (Chicago having more than Nairobi; p<0.01), the number and percentage of CD14^+^ cells (Nairobi having more than Chicago; p<0.001 and p<0.01), and the percentage of CD4^+^ T cells (Seattle and Chicago having more than Nairobi; p<0.05).

In summary, site-to-site variations in total CD45^+^ cell yields and leukocyte subpopulation distributions were minor, in particular for endocervical cytobrush and ectocervical biopsy specimens. Our study therefore demonstrates that cervical cytobrush and biopsy sampling, and the immunophenotyping of the isolated cell populations, can be performed in a standardized manner across several study sites.

### Reproducibility of cytobrush and biopsy sampling

To assess the reproducibility of cytobrush sampling, we compared the consistency of isolated immune cells from cytobrushes taken from the same participants at different times. These data were available from the Chicago site, where 12 women were sampled for both Part 1 and Part 2 of the study, with a median of 530 days between visits (IQR 503–566). Median CD45^+^ leukocyte yields across all women in Chicago were similar between Part 1 (n = 16) and Part 2 (n = 17) (12,956, IQR 6,502–29,177 vs. 15,275, IQR 4,122–38,749; p = 0.9587) (Figure 3B). Among the 12 women sampled twice, there was weak evidence of a positive correlation in CD45^+^ cell yields between Part 1 and Part 2 (r = 0.49, p = 0.1063) (Figure 3C). However, some of these 12 women showed differences of up to 10-fold between the two time points (paired samples in Figure 3B). These data suggest that cytobrush sampling yields consistent results over time when judged at the cohort level, but that individual women may exhibit substantial fluctuations.

To assess the reproducibility of cervical biopsies, two simultaneous biopsies were taken from 7 participants from the Nairobi site from the upper left and right quadrants of the ectocervix. Numbers of CD45^+^ leukocytes were strongly correlated between duplicate biopsies from the same participant-visit (r = 0.89, p = 0.0123; Figure 3D), as were the numbers of CD4^+^ T cells (r = 0.86, p = 0.0238), CD8^+^ T cells (r = 0.96, p = 0.0028), and DCs (r = 0.89, p = 0.0123; not shown). However, there were no significant correlations between numbers of CD14^+^ macrophages (r = 0.57, p = 0.2) or CD19^+^ B cells (r = 0.44, p = 0.36; not shown). Occasionally, for some subpopulations, we observed large differences between duplicate biopsies. For example, in one participant, recovery of B cells was 5,777 in biopsy A compared to 264 in biopsy B; CD4^+^ T cell recovery was similarly skewed, with 14,738 in biopsy A compared to 2,424 in biopsy B. Thus, overall leukocyte yields correlated between two ectocervical biopsies taken on the same day, but individual subpopulations sometimes differed markedly.

### Impact of blood contamination on leukocyte yields from cytobrushes

Cytobrush sampling is associated with occasional blood contamination, particularly with a second consecutive brush. At the time of sampling, clinicians recorded any visible red blood cell contamination. Using this information, we compared the cell populations obtained from cytobrushes in the absence or presence of blood. CD45^+^ cell yield from cytobrushes with blood was significantly higher than from cytobrushes without blood (median 16,760 vs. 4,603; p = 0.0078; Figure S3A). While cytobrushes with blood contamination yielded greater numbers of leukocytes, CD45^+^ cells made up a smaller fraction of all cells in those samples (median 0.49 vs. 1.4%; p = 0.0315); this may be due to the increased presence of red blood cells. There were no significant differences in leukocyte subpopulation frequencies or viabilities in the presence or absence of blood contamination (Figure S3B). The median ratio of macrophages to T cells was 0.86 in samples with visible blood and 1.05 in samples without blood (p = 0.6162).

### Impact of depot medroxyprogesterone acetate (DMPA) usage on leukocyte yields from cytobrushes

Cytobrush samples were also obtained from women at a site in Cape Town (Figure S3C). These contained higher viable CD45^+^ cell yields (median 27,866, IQR 10,291–59,386) than the other three sites for Part 2 (median 11,008, IQR 2,396–36,873; p = 0.0265), where all four sites used the same device. Approximately two-thirds (68%) of women in Cape Town used injected hormonal contraception with DMPA. In Nairobi and Chicago, DMPA use was 0% and 9% (3/33), respectively (data not available in Seattle). We hypothesized that DMPA use might explain the higher leukocyte numbers in Cape Town, and therefore compared the cell yields in Cape Town between participants on (n = 17) and off DMPA (n = 8). Leukocyte yields were similar between these groups (median 27,956 [IQR 11,683–52,380] versus 28,068 [IQR 9,040–68,190]; p = 0.9768) (Figure S3C). Thus, a preliminary conclusion of these data is that DMPA use does not explain the higher leukocyte numbers in Cape Town.

### Impact of bacterial vaginosis on leukocyte yields from cytobrushes and biopsies

Two participants (6%) from Chicago, four (27%, out of fifteen for whom vaginal Gram stains were available) from Nairobi, and 10 (32%, out of 31 for whom vaginal Gram stains were available) in Seattle, were diagnosed with BV. Eight out of 15 women participating in Part 2 in Seattle and for whom vaginal Gram stains were available had BV, enabling statistical analysis for this subset of participants. Neither cytobrushes nor biopsies showed differences in CD45^+^ leukocyte yields between women with or without BV (cytobrushes: p = 0.6943; biopsies: p = 1) (data not shown).

### Expression of α4β7 on cervical cytobrush- and biopsy-derived leukocytes

**Figure 4:**
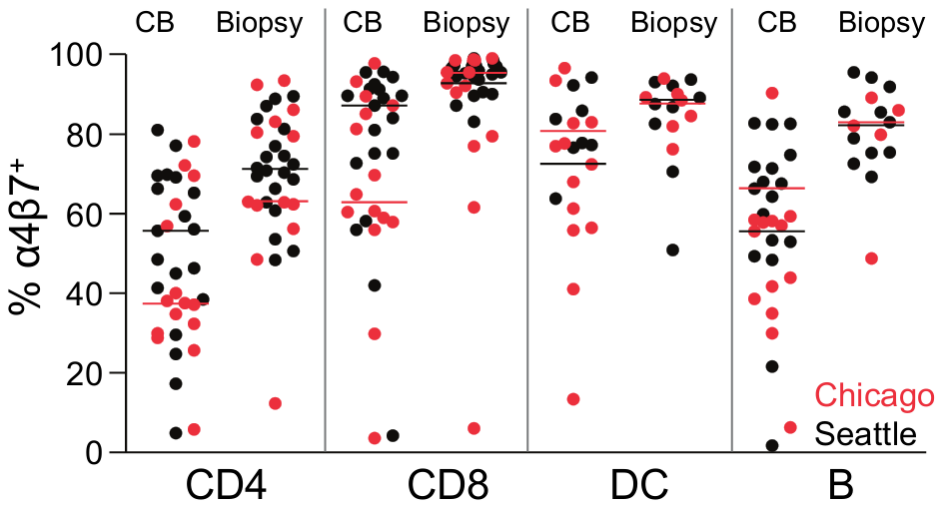
Percentages of α4β7^+^ immune cells isolated from cytobrush (CB) and biopsy samples at the Seattle (black dots) and Chicago (red dots) study sites. Percent α4β7 expression on CD4^+^ T cells, CD8^+^ T cells, CD19^+^ B cells and CD19^neg^/HLA-DQ^+^ DC. Samples are only shown if more than 50 cells were acquired for the indicated population. Horizontal bars indicate median values for each site.

The integrin α4β7, involved in homing of leukocytes to the mucosa, binds HIV-1 gp120 and previous reports have demonstrated expression of this molecule on genital tract T cells [18]–[22]. Because of its hypothesized role in promoting HIV transmission across the female genital tract [23], [24], we were interested to confirm its expression on cervical leukocytes and added an α4β7 stain to Part 2 of the study at the Chicago and Seattle sites. We found α4β7 to be highly expressed on leukocytes isolated from either cytobrushes or biopsies (Figure 4). Additionally, higher frequencies of α4β7^+^ cells were observed in biopsy-derived cells compared to cytobrush-derived cells for each subpopulation examined, including CD4^+^ T cells (median 70.9% for biopsy vs. 45.6% for cytobrush; p<0.0001), CD8^+^ T cells (median 95.4 vs. 81.3%; p<0.0001), DCs (median 84.5 vs. 72.9%; p = 0.0840), and B cells (median 80.8 vs. 55.1%; p = 0.0012). These data demonstrate high α4β7 expression on leukocytes derived from both the endocervix (cytobrushes) and ectocervix (biopsies).

## Discussion

Cellular immune responses are thought to constitute an integral part of the protective responses elicited by candidate HIV vaccines, and therefore we urgently need to better understand these at the mucosa, where HIV exposure occurs [25]. The analysis of cellular immunity requires live, functional cells, and therefore remains more challenging than measuring soluble antibodies and innate immune factors in mucosal secretions. Live mucosal cells are also required to evaluate the pharmacodynamics (cellular resistance to HIV infection) and pharmacokinetics (intracellular active drug concentrations) of candidate anti-HIV microbicides [11]. It is therefore imperative to determine the best sampling procedures to obtain viable cells from the mucosa, and optimize standardized processing and analysis protocols. This will serve to maximize achievable viable cell yields and reduce sampling variability, allowing better discrimination of biological from methodological variation [26], [27] and more reliable inter-site comparisons.

Here, we compared three common genital cell sampling methods, at two research sites in the USA and one in Nairobi, Kenya, using standardized clinical sampling, specimen processing and flow cytometric analysis procedures. Our data show that two sequential, combined endocervical cytobrushes and one ectocervical biopsy yielded a median of approximately 10,000 viable CD45^+^ leukocytes each, with the major difference lying in the relative distribution of T cells and macrophages: biopsies contained more T cells and cytobrushes more macrophages. CVL specimens consistently provide much smaller cell yields than either cytobrushes or biopsies, and are therefore not suitable for cellular analyses. Of note, in all sample types, CD45^+^ mononuclear leukocytes constituted a small minority of all cells. Most other cells were likely epithelial cells, but recently we also identified a population of neutrophilic granulocytes in cytobrushes (unpublished), which express intermediate levels of CD45 and thus were mostly excluded from our analyses, where gating was specific to CD45^high^ cells (Figures 1 and 2, Figure S1). Two previous studies also reported that neutrophils accounted for about 50% of the leukocytes in cervical cytobrushes and 10–15% in cells isolated from cervical and vaginal biopsies [28], [29].

While this result suggests that the use of biopsies is better for evaluating antigen-specific T cell responses, biopsies come with the perceived or real disadvantages of increased risk of bleeding and infection, including with HIV, as a result of the disrupted epithelial barrier [30]. In this context, the cytobrushes taken more than one year apart from the same women in Chicago, in the same stage of the menstrual cycle, are interesting. At the cohort level, cell yields and subpopulation distributions were consistent from one time point to the next, which validated the standardized procedures employed. However, in individual women, cell yields could vary up to tenfold between visits. This indicates that repeated cytobrush sampling increases the likelihood of obtaining at least one sample with a high cell yield. Since repeated cytobrush sampling is quite feasible, more frequent cytobrush sampling, and possibly pooling of cells from different clinic visits, could be a good alternative to more invasive biopsy sampling. However, for pooling to be feasible, effective cryopreservation of cytobrush cells is necessary. The Seattle authors of this paper are currently conducting systematic cryobiological studies of mucosal samples. We anticipate that improved cryopreservation techniques will soon provide more flexibility in analyzing mucosal samples, allowing shipping, banking and pooling of viable mucosal cells. Importantly, this will alleviate the need for high tech laboratories in the vicinity of each trial site, which is a major limiting factor for analyzing cellular mucosal immune responses in clinical trials.

Though cytobrushes and biopsies provide similar numbers of cells, it is notable that they are derived from two different microanatomical sites. Cytobrush sampling is performed from the endocervical canal and thus the cells are presumably derived from the transformation zone, as well as from within and beneath the columnar epithelium of the endocervix. In contrast, unless the cervix has an extensive cervical ectopy, as can occur in younger women [31], the biopsy cells will be derived from the squamous epithelium and stroma of the ectocervix. Functional differences may exist between endocervical and ectocervical leukocytes, including relative frequencies of memory and effector subsets. Whole endo- and ectocervical tissue certainly appears to contain some immunologic compartmentalization when assessed by proteomic analyses, which may impact HIV susceptibility [32]. While these differences need to be studied in more detail, it is encouraging that viable T cells and antigen-presenting cells (macrophages and DCs) can be isolated from both endocervical cytobrushes and ectocervical biopsies, likely allowing the determination of vaccine-induced cellular immune responses with either sample type. Another sample type from the female genital tract is menstrual blood, which has the advantage that it can be self-obtained by women who are instructed in the use of menstrual collection cups [33]. Mucosal cells in menstrual blood are likely derived mostly from the endometrium – it will be interesting to compare their phenotypes and function to those collected with cytobrushes or biopsies.

Consistency of sampling and analysis methods across study sites is crucial for clinical trials. Yields of viable CD45^+^ leukocytes were remarkably consistent across samples from Nairobi, Chicago and Seattle, the three sites that took part in all aspects of the study. This was particularly true for cytobrushes and biopsies in Part 2, where standardization of sampling devices and processing was rigorously implemented (cytobrush cell yields were generally lower in Part 1 than in Part 2, likely owing to some Part 1-specific issues regarding technician skill in Seattle as well as lack of sample device standardization in Part 1). The fact that the overall leukocyte yields aligned so well at the three sites validates our standardization efforts and demonstrates the feasibility of performing cellular mucosal assays in multi-site trials. It also suggests that the few differences we saw in leukocyte subpopulation distribution between sites could be driven not by methodological divergences but reflect true differences in sample composition. Likewise, CD45^+^ leukocyte yields from biopsies taken in the same procedure from two different ectocervical locations were highly correlated, but subpopulation composition sometimes varied, presumably due to regional differences between the particular tissue pieces sampled. Thus, a range of biological variables needs to be contended with when analyzing mucosal samples, which underscores the necessity for adequate sample sizes in mucosal studies.

The potential for variability in sample composition from different cohorts is underscored by our finding that cytobrushes from women in Cape Town yielded a median of 2.5 times the number of CD45^+^ leukocytes than cytobrushes from Part 2 at the other three sites. Neither blood contamination nor higher DMPA use explains this difference. It is notable by itself, though, that women using DMPA did not exhibit higher leukocyte numbers in their cytobrush samples. One of the hypotheses put forward to explain reported epidemiological evidence that women on DMPA experience higher HIV susceptibility posits that DMPA leads to higher concentrations of HIV target cells in the genital mucosa [34], [35]. Our findings suggest that DMPA use does not increase the number of HIV target cells captured by endocervical cytobrushes. However, the number of women in this particular sub-study was small, since it was only conducted at the Cape Town site, and it is unknown how sensitively alterations of cell densities in situ translate to changes in cytobrush specimens.

One of the questions repeatedly raised with mucosal samples is to what extent they are contaminated with peripheral blood. We therefore systematically analyzed cytobrushes for the impact of visible red blood cells on leukocyte yields and subpopulation distribution. We found that visibility of red blood cells increased median leukocyte yields approximately 3.6-fold, but it did not dramatically change the composition of subpopulations. The median ratio of macrophages to T cells in samples with visible blood (0.86) was comparable to samples without blood (1.05). This is in contrast to the distribution of monocytes and T lymphocytes in peripheral blood, where the average ratio of monocytes to T cells is 0.25. If visible red blood cells in cytobrushes signified substantial contamination with peripheral blood, this should have driven the ratio of monocytes/macrophages to T cells much more strongly toward the ratio generally found in peripheral blood. Thus, visible red blood cells in cytobrushes did not signify substantial contamination with peripheral blood cells, a conclusion that is supported by a previous paired comparison of blood and cytobrush cell phenotypes [28]. Rather, visible red blood cells, and concomitant leukocytes, are likely derived from local microvessels, which are actively involved in attracting leukocytes to the mucosa via specific adhesion molecules and chemokine gradients, and thus form part of the mucosa-specific environment. The same assumption applies to cervical biopsies, where visible red blood cells are nearly always observed.

The α4β7 integrin has recently been identified as an HIV binding receptor with possible pathogenetic relevance in the mucosa [18]–[22]. Approximately half of cytobrush and two thirds of biopsy CD4^+^ T cells expressed α4β7 in our study (overall range 5 to 95%), which supports the potential importance of this molecule for HIV pathogenesis. Percentages of α4β7^+^ cells were even higher among CD8^+^ T cells, B cells and DCs (macrophages could not be evaluated), but the relevance of α4β7 for HIV susceptibility is less clear for these cell types. That the α4β7 frequencies were generally higher than in past studies is likely due to the increased sensitivity achieved through amplification of Act-1 staining with two secondary reagents. Act-1 has been reported as highly specific for the α4β7 dimer [17], [22] and we performed rigorous specificity experiments for all reagents before including α4β7 staining in Part 2 of our study. Nevertheless, for the actual samples we relied on an internal negative control rather than a separate isotype control or FMO, because the study was designed to avoid splitting of samples. Thus, some uncertainty remains as to the precision of our measured frequencies, but in principle our data confirm prior publications [18], [21]. Of note, cells derived from the ectocervix expressed higher levels of α4β7 than cells from the endocervix. However, it is possible that the tissue digestion procedure might have led to up-regulation of this receptor, or that its expression decreases when cells are released from the endocervix into the endocervical canal.

In summary, we have carried out the most comprehensive study comparing cervical cell sampling methods to date, using a multi-site design to reflect the needs of global clinical trials. In addition to our data on the relative merits of cytobrushes, biopsies, and CVL, we have also provided standardized methodologies that can be used to set up multi-site studies of mucosal immunology. We have established ranges of absolute cell numbers and frequencies for each subpopulation tested. These can inform sampling decisions during HIV clinical trials. They also emphasize that even with optimal sampling and specimen processing procedures studies of genital immunity are hampered by the relatively low cell numbers available. Single cell analysis technologies, which are currently being developed with promising speed, offer the best prospect of overcoming this basic limitation [36], [37].

## Supporting Information

Figure S1 **Representative flow cytometry gating strategy for enumeration of leukocyte subpopulations.** (**A**) Forward/side scatter of endocervical cytobrush cells with depiction of Trucount bead (small box) and cell (large box) gates. (**B**) Gate used for Trucount bead enumeration. Beads gated in (A) were depicted in APC and FITC fluorescence, gated and counted. (**C**) For cell analysis, any contaminating Trucount beads were excluded using a ‘NOT’ gate. (**D**) Cells are defined as leukocytes by CD45 expression. (**E**) Viability is defined by exclusion of dead cells using the LIVE/DEAD Fixable Aqua Dead Cell Stain. (**F–H**) Identification of subpopulations of CD45^+^ viable leukocytes. (**F**) Macrophages are defined by CD14 expression and T cells by CD3 expression. (**G**) Cells defined as CD3^+^ lymphocytes in (F) are delineated into CD4^+^ and CD8^+^ T cells. (**H**) Cells gated as CD14^neg^ and CD3^neg^ in (F) are defined as CD19^+^ B cells, CD19^neg^/HLA-DQ^+^ dendritic cells, and “unknown” cells (lower left quadrant).
(TIF)

Figure S2 **Optimization of immune cell recovery from vaginal tissue for use on ectocervical biopsy in Part 2 of the study.** (**A**) Numbers of CD45^+^ cells isolated from paired samples by collagenase or enzyme cocktail digestion, or by emigration. Procedures were performed in parallel on tissue from two donors, with three replicates per procedure per donor. (**B**) Percent of recovered CD3^+^ (black) and CD14^+^ (gray) cells out of all CD45^+^ leukocytes from different procedures. (**C**) Comparison of cell numbers obtained following collagenase digestion using collagenase from Sigma (blue) and Gibco (red) in three donors. (**D**) CD4 ECD staining intensities of vaginal CD3^+^CD8^neg^ T cells after digestion of biopsies with varying concentrations of collagenase, ranging from 0.5 to 5 mg/mL (347–3470 collagen units/mL). Horizontal bars indicate medians.
(TIF)

Figure S3 **Influence of red blood cell contamination or DMPA use on immune cell yield following cytobrush sampling.** (**A**) Numbers of CD45^+^ leukocytes for cytobrush samples with or without visible red blood cells, collected at Cape Town (green), Chicago (red), Nairobi (blue), and Seattle (black). (**B**) Percentage contribution of each immune cell subpopulation to the total CD45^+^ population recovered from cytobrush samples with or without visible red blood cells. In (A–B), CBs from Part 2 at the Nairobi site and from women not on DMPA in Cape Town are excluded because the presence of blood contamination was not recorded. (**C**) Numbers of CD45^+^ leukocytes are shown for cytobrush samples from women with or without DMPA use. Only data from the Cape Town site, where many study participants used DMPA, are shown. Horizontal bars indicate medians.
(TIF)

File S1 **The full CVL processing protocol.**
(PDF)

File S2 **The full cytobrush processing protocol.**
(PDF)

File S3 **The full collagenase digestion protocol.**
(PDF)

Table S1 **Antibody panel for Mucosal cell phenotyping.** All antibodies from BD, except CD4 and CD19 (Beckman Coulter).
(DOCX)
