# Supplementary figures and images for: Optimizing Viable Leukocyte Sampling from the Female Genital Tract for Clinical Trials: An International Multi-Site Study
Source: PLoS One. 2014 Jan 15;9(1):e85675. doi: 10.1371/journal.pone.0085675 (PMC3893217; doi:10.1371/journal.pone.0085675)

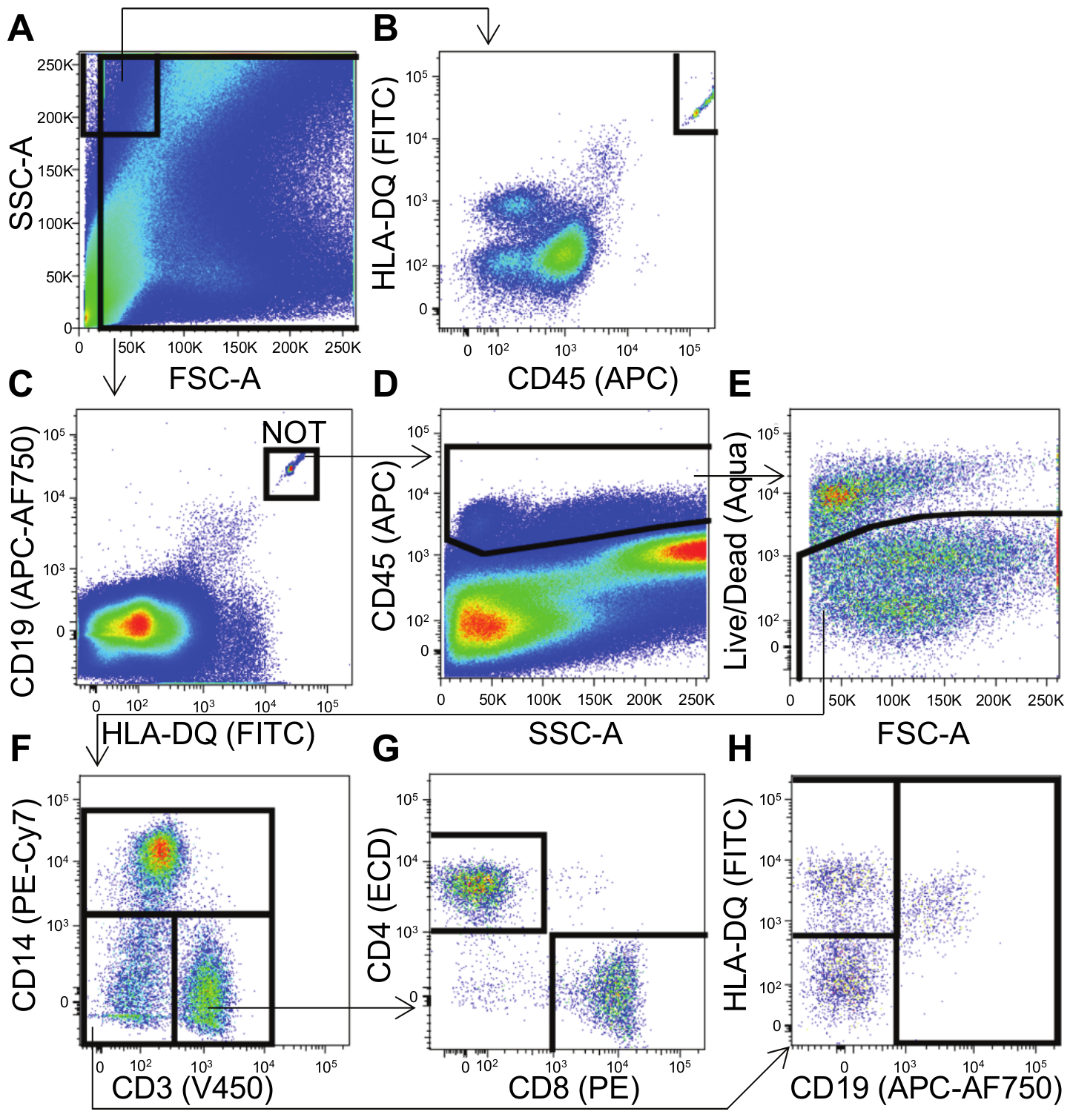

Supplement: Figure S1 — Representative flow cytometry gating strategy for enumeration of leukocyte subpopulations. (A) Forward/side scatter of endocervical cytobrush cells with depiction of Trucount bead (small box) and cell (large box) gates. (B) Gate used for Trucount bead enumeration. Beads gated in (A) were depicted in APC and FITC fluorescence, gated and counted. (C) For cell analysis, any contaminating Trucount beads were excluded using a ‘NOT’ gate. (D) Cells are defined as leukocytes by CD45 expression. (E) Viability is defined by exclusion of dead cells using the LIVE/DEAD Fixable Aqua Dead Cell Stain. (F–H) Identification of subpopulations of CD45+ viable leukocytes. (F) Macrophages are defined by CD14 expression and T cells by CD3 expression. (G) Cells defined as CD3+ lymphocytes in (F) are delineated into CD4+ and CD8+ T cells. (H) Cells gated as CD14neg and CD3neg in (F) are defined as CD19+ B cells, CD19neg/HLA-DQ+ dendritic cells, and “unknown” cells (lower left quadrant). (TIF) [file pone.0085675.s001.tif]

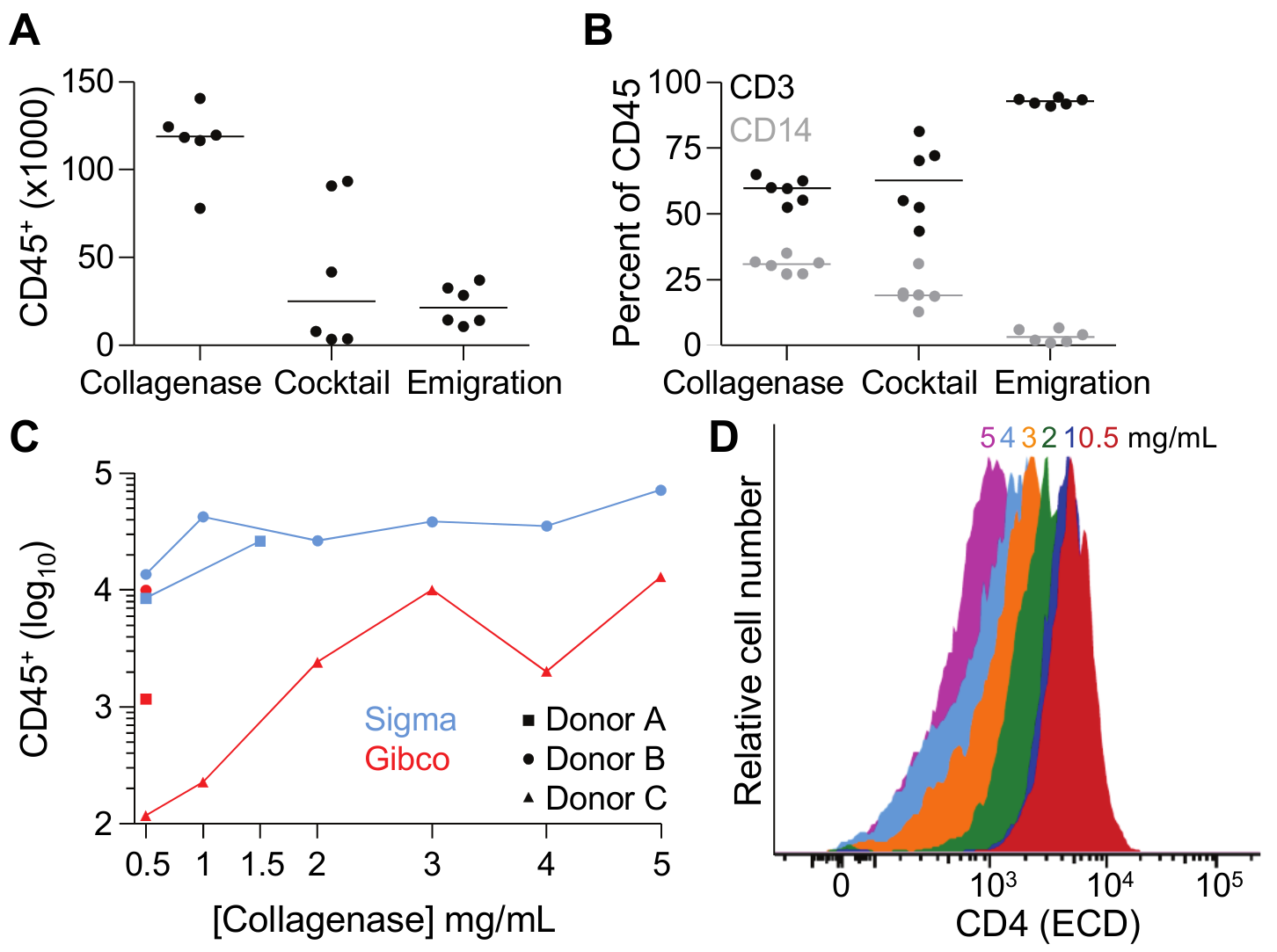

Supplement: Figure S2 — Optimization of immune cell recovery from vaginal tissue for use on ectocervical biopsy in Part 2 of the study. (A) Numbers of CD45+ cells isolated from paired samples by collagenase or enzyme cocktail digestion, or by emigration. Procedures were performed in parallel on tissue from two donors, with three replicates per procedure per donor. (B) Percent of recovered CD3+ (black) and CD14+ (gray) cells out of all CD45+ leukocytes from different procedures. (C) Comparison of cell numbers obtained following collagenase digestion using collagenase from Sigma (blue) and Gibco (red) in three donors. (D) CD4 ECD staining intensities of vaginal CD3+CD8neg T cells after digestion of biopsies with varying concentrations of collagenase, ranging from 0.5 to 5 mg/mL (347–3470 collagen units/mL). Horizontal bars indicate medians. (TIF) [file pone.0085675.s002.tif]

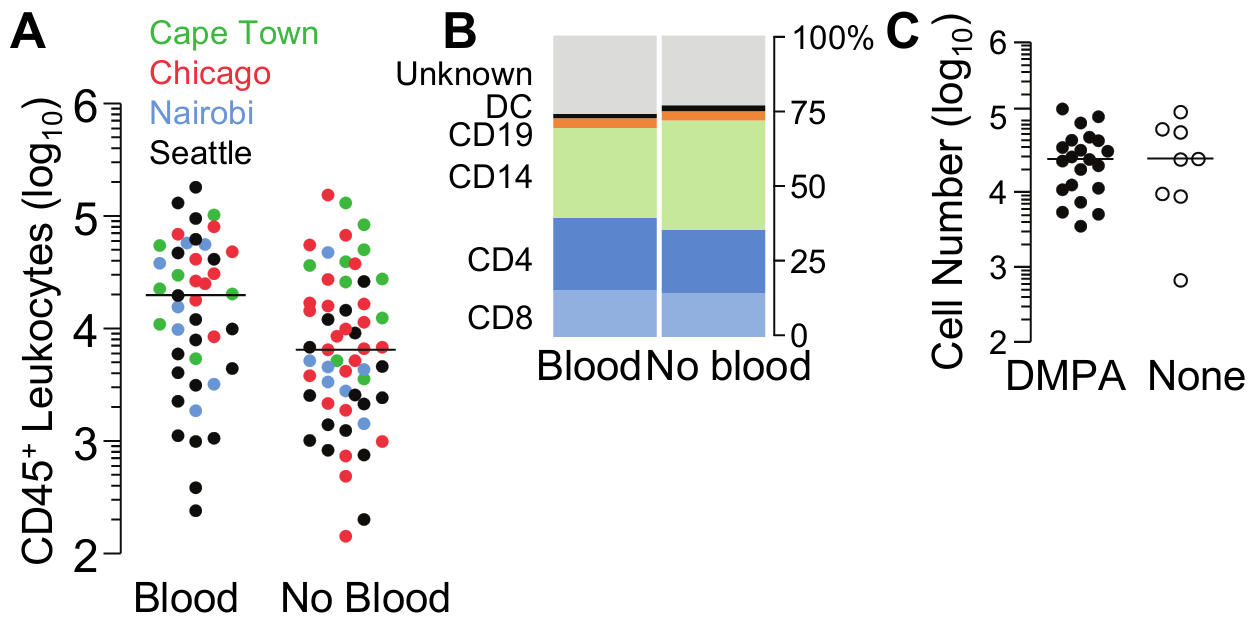

Supplement: Figure S3 — Influence of red blood cell contamination or DMPA use on immune cell yield following cytobrush sampling. (A) Numbers of CD45+ leukocytes for cytobrush samples with or without visible red blood cells, collected at Cape Town (green), Chicago (red), Nairobi (blue), and Seattle (black). (B) Percentage contribution of each immune cell subpopulation to the total CD45+ population recovered from cytobrush samples with or without visible red blood cells. In (A–B), CBs from Part 2 at the Nairobi site and from women not on DMPA in Cape Town are excluded because the presence of blood contamination was not recorded. (C) Numbers of CD45+ leukocytes are shown for cytobrush samples from women with or without DMPA use. Only data from the Cape Town site, where many study participants used DMPA, are shown. Horizontal bars indicate medians. (TIF) [file pone.0085675.s003.tif]
