# Supplementary material for: Optimizing Viable Leukocyte Sampling from the Female Genital Tract for Clinical Trials: An International Multi-Site Study
Source: PLoS One. 2014 Jan 15;9(1):e85675. doi: 10.1371/journal.pone.0085675 (PMC3893217; doi:10.1371/journal.pone.0085675)
Supplement: File S1 — The full CVL processing protocol. (PDF) [file pone.0085675.s004.pdf]

Mononuclear Leukocyte Isolation from Cervicovaginal Lavage

Procedure

1. Single Cell Preparation from Cervicovaginal Lavage
   1.1 Centrifuge the sample for 10 minutes at 250*g* (~1200 rpm on most centrifuges) with low brake.
   1.2 Carefully decant supernatant and resuspend as needed for further use.
