# Supplementary material for: Optimizing Viable Leukocyte Sampling from the Female Genital Tract for Clinical Trials: An International Multi-Site Study
Source: PLoS One. 2014 Jan 15;9(1):e85675. doi: 10.1371/journal.pone.0085675 (PMC3893217; doi:10.1371/journal.pone.0085675)
Supplement: File S2 — The full cytobrush processing protocol. (PDF) [file pone.0085675.s005.pdf]

Mononuclear Leukocyte Isolation from Endocervical Cytobrushes

Materials

1. **Cell strainer, 100µm**
   Vendor: BD Falcon
   Catalog #: 352360

2. **Centrifuge tubes, 50mL**
   Vendor: BD Falcon
   Catalog #: 352070

3. **Phosphate Buffered Saline (PBS)**
   Vendor: Gibco
   Catalog #: 14190-144

Procedure

1. Preparation
   - 1.1 Prepare a 50mL blue cap Falcon conical tube for each cytobrush.
   - 1.2 Ready a 100µm cell strainer for each cytobrush sample.

2. Single Cell Preparation from Cervical Cytobrush
   - 2.1 Place 100µm strainer in the 50mL labeled conical tube.
   - 2.2 Push a p20 tip through the hole in the cytobrush handle. Use this to remove brush from the 15mL tube.
   - 2.3 Load pipette aide with 25mL pipette.
   - 2.4 With other hand remove cytobrush from 15mL conical tube.
   - 2.5 Place cytobrush carefully on the cell strainer, continue to hold in place with one hand
   - 2.6 Use other hand to take up 20mL of room temperature PBS with a 25mL pipette.
   - 2.7 While holding pipette over cell strainer, insert cytobrush head through the 25mL pipette opening, scraping cytobrush on edge of opening as it is moved in and out.
   - 2.8 Continue to scrape the cytobrush until there are no visible remains on the brush, releasing PBS from the pipette slowly to loosen cells from cytobrush.
   - 2.9 Wash the cytobrush with another 10mL PBS. Scrape cytobrush on the strainer edge, and then discard.

2.9.1 If the cell strainer becomes clogged with mucus, use the 25mL pipette to collect the volume on the cell strainer, gently scraping the bottom of the cell strainer. Then expel the volume again. Repeat this until all the volume has drained through the cell strainer.

Note: Anytime you need to put the cytobrush down, place it in its original 15mL conical tube to prevent cell loss.

2.10 Pour the remaining 5mL media from transport tube through the strainer.

2.11 Wash the transport tube with 15mL cold PBS, then pass through the strainer, washing the strainer as well. Discard cell strainer.

2.12 If large amounts of mucus were present on brush, use a 50mL pipette to mix cell suspension gently 4-5 times to disrupt any thick mucus and release any sequestered cells. Otherwise skip to step 2.13.

2.13 Centrifuge the sample for 10 minutes at 250*g* (~1200 rpm on most centrifuges) with low brake.

2.14 Carefully decant supernatant and resuspend as needed for further use.
