# Supplementary material for: Optimizing Viable Leukocyte Sampling from the Female Genital Tract for Clinical Trials: An International Multi-Site Study
Source: PLoS One. 2014 Jan 15;9(1):e85675. doi: 10.1371/journal.pone.0085675 (PMC3893217; doi:10.1371/journal.pone.0085675)
Supplement: File S3 — The full collagenase digestion protocol. (PDF) [file pone.0085675.s006.pdf]

Mononuclear Leukocyte Isolation from Vaginal or Cervical Biopsies

Materials

1. **Cell strainers, 70 micron**
   Vendor: BD Falcon
   Catalog Number: 352350

2. **50mL conical BD Falcon tube (blue top)** (cell strainers only fit on these)
   Vendor: BD
   Catalog Number: 352070

3. **8mL polystyrene, round-bottom tube**
   Vendor Falcon
   Catalog Number: 35-2027

4. **16-gauge blunt ended needle**
   Vendor: Stem Cell Technologies
   Catalog Number: 28110

5. **3 cc syringe**
   Vendor: BD
   Catalog Number: 309585

6. **Parafilm**
   Vendor: Pechiny
   Catalog Number: PM-999

7. **Pipettes, Sterile, 2mL, 5mL, 10mL, 25mL**
   Vendor: Fisher scientific

8. **Micro Tube, Sterile, DNASE/RNASE Free 0.5 mL**
   Vendor: Fisher
   Catalog Number: 05408120

9. **Reagent Reservoir**
   Vendor: Corning
   Catalog Number: 487011.

10. **0.22 μm Stericup-GV Filter Units, 150mL, 500mL (optional)**
    Vendor: Millipore
    Catalog Number: SCGVU01RE, SCGVU05RE

11. **Cotton Gauze**
Vendor: Fisherbrand
Catalog Number: MSD14-002-50

12. **RPMI 1640 with 25mM HEPES buffer and L-glutamine**
Vendor: Gibco BRL Life Technologies
Catalog Number: 22400-089

13. **Fetal Bovine Serum (FBS)**
Vendor: Gemini Benchmark
Catalog Number: 100-106

14. **Penicillin-Streptomycin 10,000 Units**
Vendor: Gibco BRL Life Technologies
Catalog Number: 22400-089
14.1 Store at -5°C to -20°C in 5 mL aliquots.

15. **L-glutamine (100x)**
Vendor: Gibco BRL Life Technologies
Catalog Number: 25030-081
15.1 Store at -5°C to -20°C in 5 mL aliquots.

16. **D-PBS (Dulbecco's Phosphate Buffered Saline) 1x**
Vendor: Invitrogen
Catalog Number: 14190136

17. **Collagenase II**
Vendor: Sigma
Catalog Number: C6885-1MG
Lot Number:
17.1 Enzymatic activity is lot-dependent. Optimal activity is around:
Collagen: 693 units/mg
FALGPA: 2.8 units/mg

18. **DeoxyRibonuclease I (DNase)**
Vendor: Sigma
Catalog Number: DN25
Lot Number: 070M7032V
18.1 Dilute in PBS to 1U/µL and aliquot in 15 µL aliquots.

Procedure

1. Day 1 (Day prior to biopsy collection)

   1.1 Prepare R15 Media and place at 4°C.

      1.1.1 Into 500 mL of RPMI1640 with 25mM HEPES buffer and L-glutamine add 90 mL of FBS, 5 mL of L-glutamine, 5 mL of Penicillin-Streptomycin.

      1.1.2 R15 can be stored for 2 weeks at 4°C

2. Day 2 (Preparation early morning of biopsy collection)

   2.1 Confirm that temperature on orbital incubator (similar to incubators used for E. coli culture) is set at 37°C. Alternatively, a shaker water bath 2/3 filled and set at 37°C can be used.

   2.2 Prepare 13 mL fresh Collagenase Digestion Media [about 700 Collagen units/mL, generally 0.5-1.5 mg/mL, lot-specific] per subject.

      2.2.1 Remove Collagenase II vial from -20°C.

      2.2.2 Carefully measure out equivalent of 9100 units per subject.

      2.2.3 Reseal vial, store in desiccators at -20°C.

      2.2.4 In 15mL conical tube add collagenase powder, then add 6.5mL of R15, and 6.5mL of PBS.

      2.2.5 Vortex well to mix, until powder has completely dissolved, making sure no clumps get stuck on the walls or cap.

      2.2.6 Place in incubator or water bath at 37°C for 30 min prior to biopsy digestion. Note: Heating the Collagenase activates the enzyme.

      2.2.7 Before use, filter sterilize solution with 0.22 µm Millipore filter. (Optional, if cells will be placed into culture.)

      2.2.8 Collagenase Digestion Media expires one day from preparation. Always make up fresh Collagenase Digestion Media for use.

   2.3 Set out the following per subject: Two reservoirs, 3cc Syringe, 16-gauge blunt ended needle, one 70 µm cell strainer, an 8mL Falcon tube, and one 50mL Falcon conical tube.

   2.4 Label reservoirs and 50 mL conical tubes with PTID.

   2.5 Place one reservoir on gauze. This will be used to ensure that biopsies or cell suspensions do not slip out into bleach during the decanting process.

   2.6 Place second reservoir against nearest sidewall of hood. This will be used for storage of 16-gauge needle/syringe while not in use.

3. Day 2 (mononuclear cell isolation)

   3.1 If desired, cut biopsies into smaller pieces using a sterile scalpel before processing.

3.2 Wash biopsies by adding 20mL of PBS to tube containing biopsies.

3.3 Spin down 250g for 5 min at room temperature.

3.4 Take off supernatant into designated reservoir using a pipette.

3.5 Remove Collagenase Digestion Media from incubator. Clean lid with ethanol moistened gauze.

3.6 Resuspend biopsies with 3mL of Collagenase Digestion Media and transfer to labeled 8mL round bottom Falcon tube. Add 3µl of DNase (1Unit/µl).

3.7 Parafilm the lid.

3.8 Secure tubes on their sides in 37°C orbital incubator (~200rpm); tubes can be placed into zip-lock bag.

3.9 After 30min in orbital incubator remove tubes, ethanol and wipe down lid.

3.10 Remove Parafilm.

3.11 Use the syringe with a blunt-ended 16-gauge needle to break down tissue further. Aspirate and expel partially digested biopsy 10 times.

  3.11.1 Note: To avoid potential puncture incidents, place syringe loaded needle in reservoir facing towards back of hood when not in use.

  3.11.2 Note: During first couple of rounds of digestion, the biopsy may be too big to enter needle. Apply physical disruption to the biopsy with the needle tip to help break it down.

3.12 On final pass use syringe to strain the suspension through a 70µm cell strainer into a labeled 50mL conical collection tube. Be careful not to puncture cell strainer membrane with needle.

3.13 Wash 8mL round bottom tube with 5 mL R15, pouring off through strainer.

3.14 Repeat 4 times, bringing up final volume of free cell suspension to 23mL.

  3.14.1 Note: The protein in FBS inhibits the enzymes, helping to stop the reaction and preventing damage of single cells.

3.15 Place the 50 mL conical collection tube on ice.

3.16 Place cell strainer containing undigested tissue into a 6-well plate well.

3.17 Load a pipette with 3mL of fresh, warm Collagenase Digestion Media. Use this to wash cell strainer and collect tissue remaining on strainer.

3.18 Transfer the undigested tissue suspension and all 3mL of Collagenase Digestion Media to original 8mL tube for second round of digestion.

3.19 Add 3µl DNase (1Unit/µL).

3.20 Parafilm lid and place tube in orbital incubator for 30min digestion.

3.21 Centrifuge single cell suspension tube for 12 min at 250g. Ideally, centrifuge temperature should be set at 4°C.

3.22 After centrifugation of the cell suspension, take off supernatant with pipette, down to ~1 mL of media remaining, being careful not disturb the cell pellet.

3.23 Add 10mL of R15 and resuspend cell pellet.

3.24 Place tube at 4°C while awaiting other digestions.

3.25 Repeat steps 3.9-3.24 for a total of up to four incubations, straining the tissue-containing digestion media into the 50 mL conical tube each time, washing the 8mL tube and strainer with 20 mL R15 (for a total volume of 33 mL), and then centrifuging and resuspending in 10 mL.

3.26 Centrifuge cell suspension tube for 10 min at 250g and 4°C.

3.27 Decant supernatant, resuspend as needed for further use.

3.27.1 Note: Only two or three digestions may be required to dissolve the tissue completely. If any residual tissue pieces are left after four digestions, discard them.
