# Supplementary material for: Optimizing Viable Leukocyte Sampling from the Female Genital Tract for Clinical Trials: An International Multi-Site Study
Source: PLoS One. 2014 Jan 15;9(1):e85675. doi: 10.1371/journal.pone.0085675 (PMC3893217; doi:10.1371/journal.pone.0085675)
Supplement: Table S1 — Antibody panel for Mucosal cell phenotyping. All antibodies from BD, except CD4 and CD19 (Beckman Coulter). (DOCX) [file pone.0085675.s007.docx]

| Antigen | Clone | Fluorophore |
| --- | --- | --- |
| CD45 | HI30 | APC |
| CD14 | M5E2 | PE-Cy7 |
| CD3 | UCHT1 | V450 |
| CD4 | SFCI12T4D11 | ECD |
| CD8 | SK1 | PE |
| CD19 | J3-119 | APC-Alexa 750 |
| HLA-DQ | SK10 | FITC |

**Supplementary Table 1. Antibody panel for**

**Mucosal cell phenotyping.** All antibodies from

BD, except CD4 and CD19 (Beckman Coulter).
